# Feasibility of Minimally Invasive Robot‐Assisted Retrofacial Access to the Stapedius Muscle for Applications in Objective Cochlear Implant Fitting: A Cadaveric Study

**DOI:** 10.1002/rcs.70178

**Published:** 2026-05-04

**Authors:** Dirk Arnold, Francesca Maule, Pablo Galeazzi, Mohannad Al‐Qubaj, Orlando Guntinas‐Lichius

**Affiliations:** ^1^ Department of Otorhinolaryngology Jena University Department Jena Germany; ^2^ MED‐EL Medical Electronics Innsbruck Austria

**Keywords:** anatomic landmarks, cochlear implants, electrical stimulation, fitting, minimally invasive surgical procedures, reflex, robotic surgical procedures, stapedius

## Abstract

**Background:**

Accurate setting of maximum comfort levels (MCL) during cochlear implant (CI) fitting is essential but subjective, especially in non‐cooperative patients. The stapedius reflex (SR) correlates with the MCL and offers an objective measure. Surgical access to the stapedius muscle (SM) is challenging due to its proximity to the facial nerve and sigmoid sinus; a retrofacial approach is preferred. This study evaluated the feasibility of robotic‐assisted SM access via this approach.

**Methods:**

Two cadaveric heads underwent robotic drilling using the HEARO system. Preoperative CT imaging and 3D planning with OTOPLAN defined patient‐specific trajectories to the SM while maintaining safe distances from critical structures. Accuracy was assessed with postoperative CT and microscopic dissection.

**Results:**

The SM was successfully achieved in both specimens with high precision and minimal invasiveness.

**Conclusions:**

Robotic‐assisted retrofacial access to the SM is feasible and safe, supporting future precision surgery and closed‐loop CI systems with objective SR monitoring.

## 1 Introduction

The cochlear implant (CI) is widely successful in restoring auditory perception in individuals with severe‐to‐profound sensorineural hearing loss [1]. Ensuring consistent and optimal hearing with the device depends on the setting of the correct levels of electrical stimulation in a process called fitting. Fitting is of paramount importance for the CI to achieve its clinical efficacy and for the CI user to achieve the best possible hearing experience [2].

The fitting process relies on defining the most comfortable loudness levels (MCLs) of stimulation based on the CI user's subjective perception, that is, expressing their discomfort when the stimulation is too high. As such, using this method intrinsically carries some limitations in terms of repeatability, reliability and sometimes feasibility, particularly in cases with non‐cooperative CI users. This can often be the case in young children or users with additional needs.

The activation of the stapedius muscle (SM) through the stapedius reflex (SR), also referred to as the acoustic reflex, is known to correlate closely with the MCLs in CI users [3]. The SR is evoked involuntarily as a type of safety mechanism, which limits the movement of the stapes in response to loud sounds to protect the inner ear, or in cases with CI use in response to excessive electrical stimulation levels. Therefore, the potential to determine SM activity represents a significant advancement in establishing reliable, patient independent objective and adaptive fitting of CIs.

We previously investigated the feasibility and safety of accessing the SM via a retrofacial approach to directly record its activation via electromyography (EMG) [4]. Access was successfully achieved during standard CI implantation surgeries and SM‐EMG was collected in response to both acoustic and electrical stimulation of the SR (paper in preparation for submission). Despite proving the safety and reproducibility of accessing the SM via this approach, we observed that the proximity of the SM to critical anatomical structures, such as the facial nerve (FN) and the sigmoid sinus (SS), may lead to CI surgeons being reluctant in choosing this approach. A potential solution to overcome the difficulty of accessing the SM via the retrofacial recess is the use of robotic assisted surgery (RAS) techniques.

Since its emergence in the late 1980s and early 1990s, RAS has become a valuable technique used in many different fields, including urology, gynaecology, and head and neck surgery [5, 6, 7, 8, 9]. The da Vinci system was the first surgical robot to receive FDA approval after being designed for trauma surgery in military conflict zones. It paved the path for the use of robotics in standard operating rooms [6]. Since 2005, the Da Vinci has been approved to perform transoral resections of head and neck tumours [8]. Through transoral robotic surgery (TORS), robotic surgery has emerged in the fields of otolaryngology and head and neck surgery. Otology is well suited for robotics because of the need for precision in small, rigid spaces in the temporal bone. RAS can navigate the complex anatomy, allows for a reduction in invasiveness, and can overcome hand tremor. This can enhance precision using three‐dimensional (3D), high‐definition magnified views combined with ‘wristed’ instruments that can move beyond the range of human hands to facilitate manoeuvres in the limited space of the ear and improve consistency [6, 10, 11, 12]. Thus, the latest innovation in CI surgery is robot‐assisted cochlear implantation surgery (RACIS). Using RACIS, the HEARO robot creates a minimally invasive keyhole trajectory to the cochlea, thereby avoiding the *conventional* extensive mastoidectomy and posterior tympanotomy typically required to expose the facial recess [13]. This requires optimal planning of the access route [14], 3D reconstruction of temporal bone imaging data, and the use of planning software (OTOPLAN) to define the access in relation to critical neighbouring structures, such as the facial nerve (FN) [15]. To date, SM has not been of interest in RAS.

Preliminary steps to support RAS to plan the access to the SM were accomplished in an earlier work of ours [16], where the anatomical configuration to access the SM in relation to the FN was investigated and a surgical approach based on a 3D shape termed *operational cone* was designed. The operational cone included all the linear trajectories that could be used by RAS to reach the SM from the surface of the temporal bone (TB), via the retrofacial approach. The surgical planning tool showed that a retrofacial access to the SM is feasible in 83% of cases and this was confirmed in 13 standard CI surgeries.

Moving forward, the present study aimed to determine, given the suitability of RAS in otology, if the SM could be reached via the retrofacial approach using HEARO robotic drilling via a linear trajectory. To determine the feasibility of the use of robotics in this approach, cadaveric temporal bones were used and an image‐guided, semi‐automated, robotic platform specifically designed for minimally invasive cochlear implant surgery was developed.

## 2 Methods

### 2.1 Study Design and Samples

In this prospective, single centre study, two alcohol glycerin conserved human cephalic specimens were used for RAS with permission from the Medical University of Innsbruck, Department of Anatomy, Histology and Embryology, Institute of Clinical and Functional Anatomy.

The temporal bones of the heads from two different donors, one left and one right temporal bone, were drilled using an image‐guided, semi‐automated, robotic platform specifically designed for minimally invasive CI surgery (HEARO, Cascination AG, Bern, Switzerland). Full heads were employed and no dissection to extract the temporal bones was performed.

### 2.2 Imaging

To begin, computed tomography (CT) images of the samples were acquired by means of a C‐arm CBCT system (XCAT XL, Xoran Ltd., Ann Arbour, Michigan, USA) with a resolution of 0.1 mm isotropic voxel size. Semi‐automatic segmentation of the key structures, including the mastoid segment of the FN, posterior semicircular canal (PSC), pyramidal eminence (PE), and SM, was performed via the 3D Slicer software [17] to preliminary evaluate the anatomy of the area surrounding the SM. The feasibility of the retrofacial approach was assessed according to Volk et al. [18].

#### 2.2.1 Preoperative Plan (Linear Trajectory Computation)

A 3D surgical planning tool (OTOPLAN, Cascination AG, Bern, Switzerland) was then used to reconstruct the underlying structures and enable precise preoperative plan computation, namely, the definition of a linear trajectory to the SM while maintaining defined safety distances from critical structures (see below).

To plan the linear trajectory the data collected was first processed in OTOPLAN using the following steps:

1. Reconstruction of the bony tissue of the temporal bone with semi‐automatic thresholding
2. Identification in a stepwise manner, by means of manually placed points on the CT scan, the FN, the SS, and the ossicular chain (OC), and their semi‐automatic reconstruction.
3. Manual setting of the final target point on the belly of the SM. The correct position was identified by an experienced otosurgeon (O.G.L.).
4. The trajectory was defined by running a preliminary trajectory from the external surface of the temporal bone to the target point using the software. The result was then adjusted manually via stepwise fine tuning (in both the anterior‐posterior direction and the superior‐inferior direction) to reach a safe retrofacial access to the SM (optimal trajectory). The minimum safety distance respected default values of 0.4 mm distance to the FN and 0.3 mm distance to other critical structures, including the chorda tympani, external auditory canal, sigmoid sinus (SS), stapes (S), incus, and malleus.
5. Once the optimal trajectory was established, the drilling depth was set to safely reach the SM by again complying with the aforementioned drilling‐safety parameters.

### 2.3 Surgical Procedure

After the pre‐operative plan computation, the surgical procedure was performed via the following steps:

1. Specimen Preparation: the specimen head was draped and positioned in the HEARO system headrest.
2. Incision and Fiducial PlacementA postauricular incision was performed to expose the cortical bone. Five titanium fiducial screws were inserted into the mastoid cortex as artificial landmarks to enable patient‐to‐image registration. To allow correct placement of the fiducial screws, the potential surface entry region for the planned drilling trajectory was first identified on the mastoid cortex. At this preparatory stage, the objective was not to determine the exact geometric entry point of the final linear trajectory but rather to outline a sufficiently constrained sub‐region on the bone surface around which the fiducials could be positioned. For this purpose, the surgeon replicated the linear distance between the lateral extremity of the external auditory canal and the trajectory entrance point derived from the pre‐operative OTOPLAN plan. In addition to this distance cue, the surgeon used visual anatomical assessment including mastoid surface orientation and EAC orientation to estimate the approximate projection of the stapedius muscle on the cortical bone. This qualitative localisation was adequate; the approximate marking at this stage served solely to ensure that the fiducial screws circumferentially surrounded the future drilling corridor and did not influence the accuracy of the final robotic trajectory.
3. Imaging: a new CBCT scan was acquired with the fiducial screws in place.
4. Image Fusion and Plan Transfer: the new CT dataset embedding the fiducial screws was imported into OTOPLAN and automatically co‐registered with the dataset used for the preoperative plan computation. Fiducials were automatically detected and modelled by the software, and the previously computed surgical plan was confirmed before being exported to the HEARO system.
5. Patient to image co‐registration: the HEARO patient marker was attached to one fiducial screw, and the remaining four screws were used for patient‐to‐image registration, allowing the system to identify exactly the position in the physical environment of the sample to be drilled.
6. Drilling: a 1.8 mm diameter drill bit was used to create a minimally invasive tunnel from the mastoid surface to the pyramidal eminence (PE) region. Drilling was executed under robotic control, with activation supervised by the surgeon. The procedure consisted of:
  ◦ Retrofacial mastoid drilling phase: drilling through the mastoid towards the SM target point at a controlled feed rate (0.5 mm/s) and rotation speed (1000 rpm).
  ◦ Fine Milling phase: ultimate drilling phase with a 1.0 mm burr used to refine access to the SM.

### 2.4 Anatomical Validation and Accuracy Evaluation

Once the surgical plan was accomplished, a surgical rod was placed into the drilled corridor and new CT images were acquired for post drilling segmentation using 3D Slicer software and accuracy evaluation using OTOPLAN software. The following imaging, the temporal bones were dissected under a surgical microscope to confirm exposure of the SM via the retrofacial approach. The drilled tunnel was identified using a reference rod. Photographic documentation was collected.

## 3 Results

The present study verified the feasibility of accessing the SM via the retrofacial approach using robotic assisted drilling. The drilling trajectory was linear starting from the external surface of the temporal bone, passing anterior to the sigmoid sinus and retrofacial to the facial nerve, until reaching the belly of the SM.

The acquisition of the CT images from the two cadaveric samples allowed a preliminary assessment of the feasibility of the retrofacial approach by evaluating the relative spatial configuration between the SM and the FN.

**FIGURE 1:**
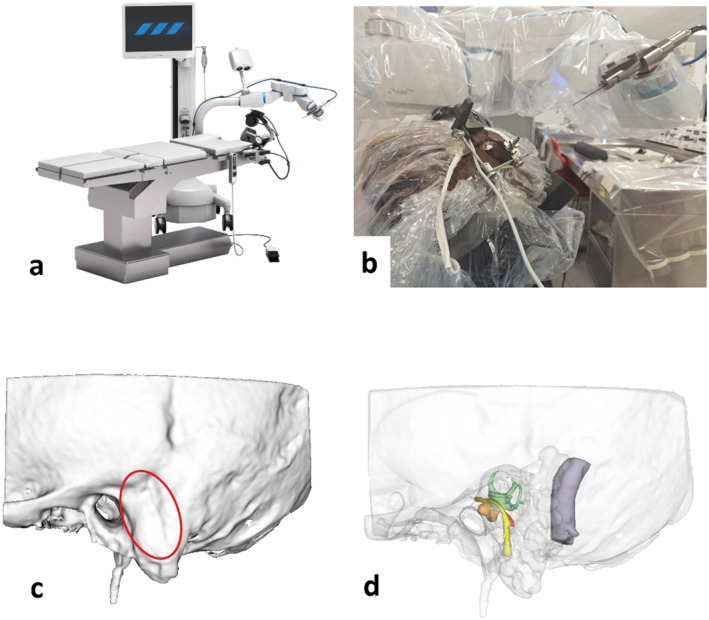
Depiction of (a) the HEARO robotic system used in this study. In the present study, only the cadaveric head was used in the system. Panel (b) shows a picture taken during the drilling session depicting the specimen placed in the headrest (right case). On the top right corner, the drilling element of the Hearo system approaching the head is visible. Panel (c) shows the three‐dimensional reconstruction of the left temporal bone; a red oval marks the targeted area of the drilling session; and panel (d) shows the anatomy of the targeted area. Yellow = facial nerve, lilac = sigmoid sinus, light orange = cochlea, green = vestibular organs, red = stapedius muscle.

Figure 1 shows the configuration of the HEARO robotic system, the drilling setup, the targeted drilling area on the temporal bone, and the underlying anatomy.

**FIGURE 2:**
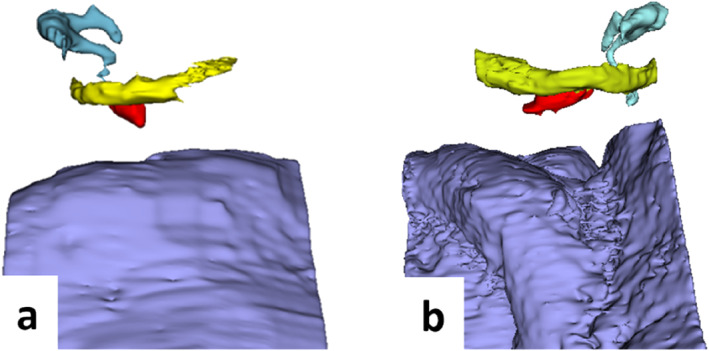
Three‐dimensional reconstruction showing the relative position of the stapedius muscle and the facial nerve from the cadaveric specimens drilled in the study (coronal perspective). Panel (a) shows the right side of one of the specimens drilled, while panel (b) shows the left side from the other specimen drilled. The segmented structures are coloured as follows: red = stapedius muscle, yellow = facial nerve, light blue = ossicular chain, lilac = sigmoid sinus.

The 3D reconstructions of the two drilled anatomies are shown in Figure 2. In both cases, the SM appeared to be exposed with respect to the FN, and the SS did not show any excessive prominence that could cause obstruction to a potential linear trajectory. Thus, the anatomy in both cases was suitable for the retrofacial approach. The linear trajectory to access the SM was computed in OTOPLAN, after semi‐automatic segmentation of the crucial anatomical parts involved by manually placing the final target on the belly of the SM manually. The safety of the planned procedure was respected by compliance with the drilling‐safety parameters.

**FIGURE 3:**
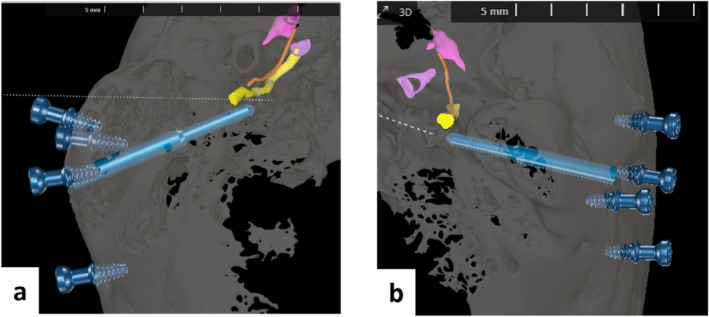
Three‐dimensional reconstruction of the CT scan in OTOPLAN and the simulated linear trajectory to access the stapedius muscle retro facially for from both cadaveric specimens (axial perspective). (a) right side, (b) left side. The segmented structures are coloured as follows: yellow = facial nerve, orange = chorda tympani, lilac = sigmoid sinus, pink = incus, light blue = simulated drilling bit along the linear trajectory, dark blue = fiducials screws. The target on the stapedius muscle belly is represented by a pink circle.

The 3D reconstruction of the CT scan in OTOPLAN and the simulated linear trajectory to access the SM retrofacially for both temporal bones are shown in Figure 3.

**FIGURE 4:**
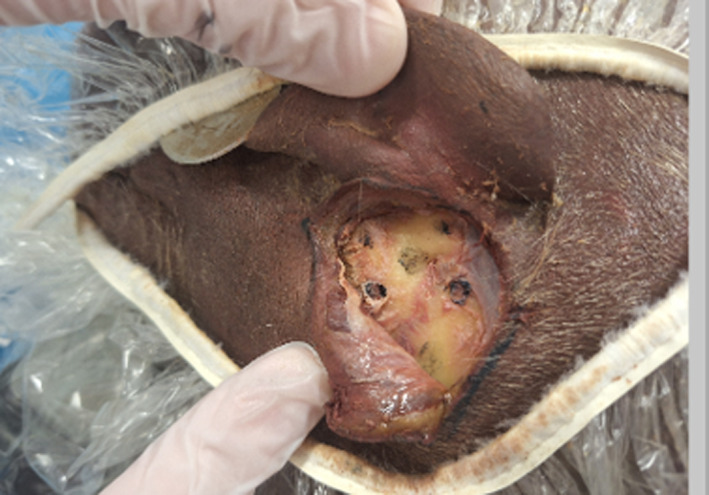
Sample preparation on the left side of one specimen. The pinna is flipped anteriorly. The four holes in the temporal bones are the placement holes for the fiducial screws. The target entry point in the temporal bone for the computed drilling trajectory is shown by a star.

The initial step of the sample preparation on the left side of one specimen is presented in Figure 4. An incision was made behind the ear of the cadaver, and the pinna was flipped anteriorly. The screws were positioned around the targeted entry point of the drilling bit on the temporal bone.

**FIGURE 5:**
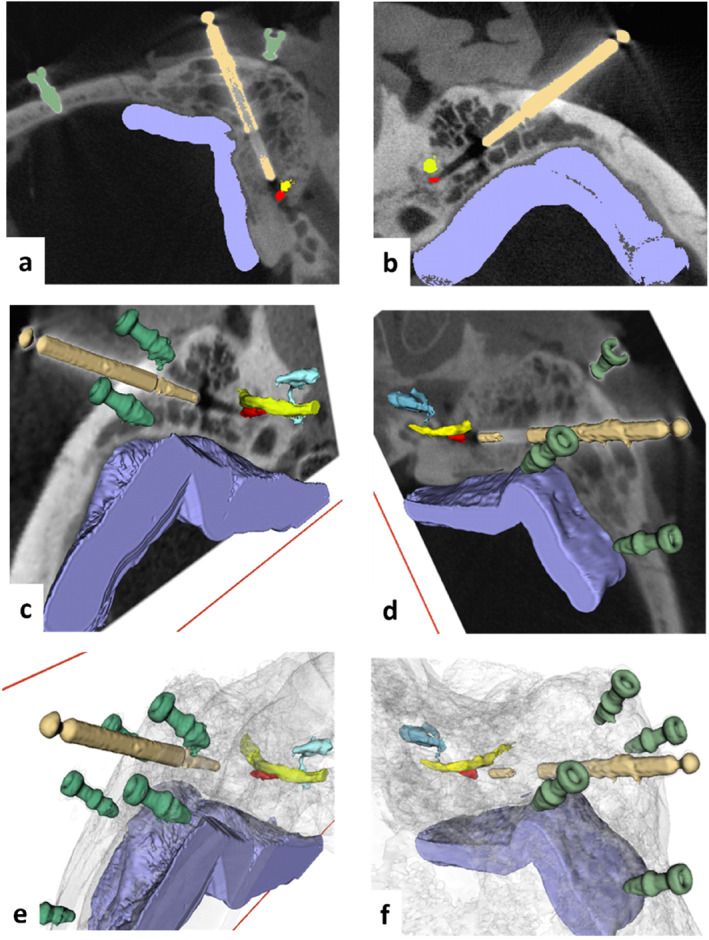
Results of the drilling session. Panels (c), and (e) show the results from the left side. Panel (b), (d) and (f) are the results from the right side. Panels (a) and (b) show the linear trajectory reaching the stapedius muscle. Panels (c) and (d) show the same CT slices of (a) and (b) overlayed on the 3D post‐drilling reconstructions. In panels (e)and (f), the bone is overlayed on the 3D reconstruction to give a complete overview. The segmented structures are coloured as follows: beige = surgical rod inserted for the post‐surgical CT scan; red = stapedius muscle; yellow = facial nerve; lilac = sigmoid sinus; green = fiducial screws; and light blue = ossicles.

The final results of the drilling session in both cases are presented in Figure 5. The rod was not inserted fully to the end of the drilled corridor, which proceeded in depth until the belly of the SM, leaving the FN and the SS intact.

The accuracy of the drilling distances with respect to the facial nerve and the sigmoid sinus, and the error in target achievement are reported in Table 1 and Table 2.

**TABLE 1:** Accuracy of the drilling distances with respect to the facial nerve and the sigmoid sinus in both specimens.

	3150R			4027L		
Closest distance to the following anatomical structure (mm)	Preop	Intraop	Δ (intraop–preop)	Preop	Intraop	Δ (intraop–preop)
Facial nerve	1.78	1.81	0.03	0.93	1.30	0.37
Sigmoid sinus	1.84	1.75	−0.09	5.73	5.57	−0.16

**TABLE 2:** Error in target achievement to hit the stapedius muscles in both specimens.

	3150R		4027L	
	Entrance	Target	Entrance	Target
Error F (mm):	0.08	0.13	0.16	0.59

**FIGURE 6:**
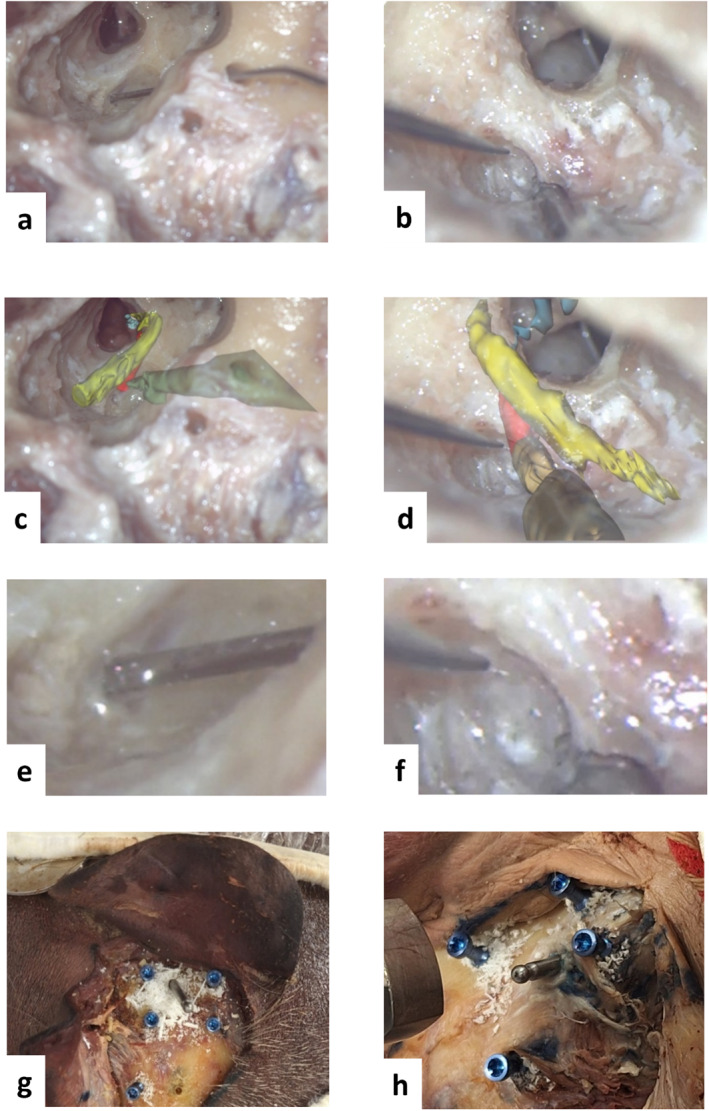
Post drilling dissection after the retrofacial approach until exposition of stapedius muscle tissue on the left (L) and right (R) sides. Panels (a) and (b) show a microscope view of the dissection. The linear trajectory drilled with the robot is identified by the needle inserted along the channel. Panels (c) and (d) show the same view from the microscope overlayed with a transparent 3D post‐drilling reconstruction of the specimen. Panels (e) and (f) show the magnified view of the final drilling point and a small section of the uncovered muscle tissue, and panels (g) and (h) show the final view of the cadaveric specimen after the drilling. A surgical rod was placed to identify the surgical corridor inside the drilled trajectory. The fiducial screws are shown in blue. The segmented structures are coloured as follows: red = stapedius muscle, yellow = facial nerve, light blue = ossicular chain, green = surgical rod.

Further evaluation of the drilling success was performed via dissection of the drilled specimens. Figure 6 shows the post drilling dissection on both sides of the specimen. After drilling the mastoidectomy, the surgeon proceeded retrofacially until reaching the SM tissue. The linear trajectory drilled by the robot was identified by a needle inserted along the drilled channel. A small section of the SM tissue was left uncovered on both sides. A surgical rod was placed to identify the surgical corridor inside the drilled trajectory.

## 4 Discussion

This study demonstrated that the SM could be accessed successfully via the retrofacial approach using the HEARO robotic system with a minimally invasive, pre‐planned linear trajectory.

This development in robotic surgery represents a promising step towards improving the safety of accessing the stapedius muscle via the retrofacial approach. In our earlier work, we have shown that the SM can be accessed safely and reliably in 81.2% of cases [4]. However, given that the SM is a structure totally embedded in the bone and running close to the FN, accessing it frequently represents a challenge to otosurgeons, with the risk that critical structures may be damaged while drilling in such close proximity to the FN. Robotic drilling addresses this challenge by offering enhanced precision [10, 19, 20] and a standardised approach to the execution of surgery that is also minimally invasive.

Intraoperative accuracy with respect to the pre‐operative planned trajectory has been shown to reach the order of a fraction of a mm (0.182 mm to target and 0.117 mm to facial nerve) [19]. In the context of our study the data showed that in one case the accuracy was comparable (0.13 mm to target and 0.03 mm to FN) while in the second case the accuracy was lower (0.59 mm to target and 0.37 mm to FN). Given the limited sample size, one would expect that occasional deviations from the optimal accuracy may occur, and this should be considered within the context of the study. Our findings should be interpreted as complementary rather than definitive for assessing the overall success of this feasibility trial. Another aspect to consider regarding such observed variability is that between the two specimens might reflect the cadaveric feasibility‐study setting, where several workflow safeguards routinely applied in clinical robotic procedures (e.g., re‐registration after any marker disturbance) were intentionally not implemented, as accuracy was not the primary study objective. In a clinical setting, unintentional movement of the patient marker or disruption of the tracking geometry would prompt immediate corrective actions to preserve spatial accuracy, whereas during this cadaveric evaluation, the workflow was not optimised to that degree. Differences in specimen fixation, mechanical stability, and user interaction during the procedure may therefore contribute to the observed variability and definitely represent aspects that need optimisation for future studies. Moreover, it is important to clarify how the accuracy values in Tables 1 and 2 are derived. These values do not represent the ability of the CBCT system to resolve anatomical structures below the imaging resolution. The CBCT acquisition used in this study produced 0.1 mm isotropic voxels, meaning that sampling was uniform in all three spatial dimensions but still limited by the intrinsic spatial resolution of the system. According to the Nyquist sampling criterion, the smallest reliably resolvable anatomical detail is approximately twice the sampling interval (∼0.2 mm). Anatomical separations smaller than this cannot be physically discriminated by the imaging system. However, the accuracy values reported here arise from a different process: they are calculated by comparing the predicted trajectory (planned on the pre‐operative CBCT containing four fiducial screws) with the actual trajectory measured on a second CBCT acquired after drilling, in which the same fiducials and a reference rod were present. Because both volumes share identical fiducials, the rigid registration between them achieves sub‐voxel numerical stability, independent of the anatomical resolution. Consequently, the sub‐0.2 mm values reported (e.g., Δ = 0.03 mm or entrance accuracy of 0.08 mm) reflect geometric deviations within the planning–registration–drilling workflow, rather than true anatomical measurement accuracy. These values should therefore be interpreted as indicators of system consistency and robotic execution precision and not as anatomical distances resolved from the imaging data.

One must also consider when using the robotic approach that the computed surgical trajectory is patient‐specific, which means that the drilling pathways are planned on real anatomical CT data from the patients. Therefore, the patient specific SM structure, its relative position to the FN, the size of the SS, and the air cell structure are all quantitively assessed during the trajectory pre‐planning and incorporated into the target definition plan to minimise any potential ‘trial and error’ of target identification [21, 22]. This is of particular importance when the target region is not visible to the surgeon, as in the case of the SM, because by means of quantitative 3D segmentation, concealed anatomical structures can be mapped in terms of their shape, size, and spatial position.

A second positive aspect of this approach is that the procedure is less invasive using robotic drilling. When performing the manual retrofacial approach to expose the SM, the surgeon is required to remove several layers of tissue to reach a depth below the FN; this—in addition to the extra time required for drilling ‐ implies the treatment of a wide area in the temporal bone, and an increased probability of interfering with or damaging critical structures such as vessels, and vestibular organs. In contrast, the robotic trajectory allows access to the SM through a substantially narrower and more focused drill path. We highlight here that our comparison in terms of time performance refers solely to the traditional retrofacial approach for SM exposure and no data about direct comparison between the procedures (CI implantation + retrofacial SM access) robotically performed with HEARO or manually performed are available to our knowledge. Another aspect to consider is that manual retrofacial drilling demands noteworthy cognitive processing due to the anatomical complexity of the region, requiring surgeons to integrate their detailed anatomical knowledge with continuous identification of critical structures, some of them embedded in bone throughout the procedure.

In contrast, the robotic drilling, which in our case was performed in a linear corridor of about 1.8 mm diameter, drastically reduces the invasiveness of the approach and the necessity to expose the mastoid excessively [6]. This potentially minimises tissue damage, improves the rate of recovery, reduces the risk of infection, and improves the patient experience.

Furthermore, in our experience, the robotic approach also increased the likelihood of reaching the final drilling target point. Although a surgical approach performed via a drilled channel (and not a wide dissection) does not offer a clear visibility of the surgical field and of the final drilling target point, it allows the final target point to be reached with precise targeting via the surgical planning tool and the semi‐automated drilling guidance. Furthermore, it automatically creates a naturally guided trajectory for a possible electrode insertion, limiting the occurrence of wrong placement and fostering reproducibility of the procedure [15, 20].

A further advantage supporting the system's performance are the sensors integrated into the robotic system that monitor the mechanical forces/torques and heat damage. In robotic surgery, the lack of force feedback is often a challenge for the surgeons to maintain precision during the procedure while at the same time preserving tissue [23]. Such sensors contribute to the efficient protection of the critical anatomical structures, from thermal or mechanical damage. The operator tremor is also controlled using the robotic system and facilitates more accurate access [24]. Using a keyhole approach to access structures in the facial recess means that drilling is carried out in the vicinity of the facial nerve at distances as close as 0.5 mm. The visibility of the facial nerve itself is reduced; however, having the potential to monitor the facial nerve activity and preserve it via facial nerve monitoring units embedded directly into the drill, offers crucial additional feedback. Taken together with the other aforementioned features, it contributes to improved surgical safety overall [25].

By limiting the risks associated with surgery and simplifying the procedure, it brings us a step closer to the possibility of creating ‘closed‐loop’ smart CI systems in the future. Present day CI systems are considered ‘open‐loop’ because they require extensive fitting immediately after implantation and at regular intervals to maintain optimal performance [26]. A closed‐loop system would require that the patients maximum comfort level (MCL) could be defined without the need for subjective feedback. The MCL is used in CI fitting because it is the highest electrical stimulation level that a cochlear implant patient perceives as comfortable when using their CI. The MCL has been shown to be correlated with the electrically evoked stapedius reflex [27, 28, 29]. Thus, with access to the SM, the estimation of the MCL can potentially be achieved by means of the electrically elicited stapedius reflex to enable real time monitoring of the muscle's activity and the automatic adjustment of stimulation levels in response in the future.

Despite the step forward our study has achieved in accessing the SM via a retrofacial approach using robotic surgery, there were a number of study limitations. Firstly, the small number of procedures which were performed in cadaveric heads. Therefore, the data is preliminary and more data are needed to corroborate the feasibility we observed herein. Another factor to take into account is that the robotic drilling relies considerably on accurate pre‐operative images and exact segmentation to identify the patient's anatomical structures and one must ensure that errors or variability in segmentation are not a limiting factor to safety and precision in clinical practice [21, 22].

Another aspect to consider is the learning curve. The learning curve in robotic surgery is inherently multifactorial and can vary considerably for several reasons. Beyond individual differences in the acquisition of surgical proficiency, an important determinant is the level of surgeon involvement in the actuation of the procedure. Telemanipulated systems (e.g., the Da Vinci platform) rely on real‐time, continuous instrument control by the surgeon, whereas semi‐autonomous systems such as HEARO perform pre‐planned drilling, with the surgeon assuming a primarily supervisory and decision‐making role rather than executing every movement manually. These fundamental differences influence not only the nature of the skills required but also the time needed for personnel to become proficient [30]. As a result, the duration and shape of the learning curve may vary substantially between individuals, applications, and robotic platforms, making learning‐curve estimation a crucial—and non‐negligible—multifactor aspect of robotic surgery implementation in transoral robotic surgery, it has been shown that learning curves may peak at as many as 20–30 cases; however, it is surgeon specific [31] Another aspect, worthy to be discussed is the radiation exposure to which a potential patient receiving our procedure needs to undergo. The workflow in fact requires multiple CBCT acquisitions—typically including a preoperative scan for anatomical planning, an intraoperative scan with fiducials in place for registration, and a postoperative scan to verify electrode placement. Although each individual acquisition delivers a relatively low dose compared to conventional CT, the cumulative exposure might be non‐negligible and represents a trade‐off inherent to the method. This aspect is particularly relevant in the context of a procedure presented as minimally invasive. Therefore, dose‐reduction strategies can help mitigate such drawbacks. At last, implementing robotic systems in ENT is expensive. The surgical robots and their maintenance can represent a six‐figure investment [32]. Moreover beyond the initial investment, longer operative times may contribute to overall cost, particularly when compared with conventional cochlear implantation. In our case, it is important to clarify that the time comparison refers specifically to the duration of the retrofacial approach for stapedius muscle exposure (manual vs. robotic access) and not to standard CI surgery. In this specific context, the robotic retrofacial access was shorter than the manual technique. Nonetheless, for broader clinical applications of robotic systems, increased setup and workflow duration may influence costs and resource utilisation. To our knowledge, large studies that compare robotics to conventional surgery and evaluate these effects do not exist to date, as RACIS is a relatively new intervention. Studies are needed to directly compare the benefits of robotic surgery, in terms of complication rates, relative efficiency in terms of surgery time, and their auditory outcomes [20, 33]. These are important factors going forward that were not considered in this cadaveric study. Nonetheless, our promising but still preliminary results—despite not including a direct comparison with a manual manual drilling group ‐ indicate that the robotic approach offers several promising advantages for this technically demanding procedure, although these findings must be interpreted cautiously given the limited sample size.

## 5 Conclusion

This study has demonstrated the feasibility of accessing the SM via a minimally invasive, patient‐specific linear trajectory using a robotic system. Through enhanced surgical precision and reduced anatomical disruption, robotic drilling addresses key challenges associated with accessing the SM via the retrofacial approach manually. Safety is improved because the robotic system integrates preoperative imaging, quantitative segmentation, and sensor‐based feedback mechanisms, which improve surgical consistency and the predictability of a safe, and targeted surgical outcome. This facilitates future research and development into closed‐loop CI systems, which will pave the way for a more patient orientated CI fitting strategy.

## Author Contributions

O.G.L. conceptualization. D.A., F.M., P.G., and M.A.Q. methodology. O.G.L. and F.M. validation. F.M. formal analysis. D.A. and O.G.L. investigations. P.G. and M.A.Q. resources. F.M. data curation. F.M. and O.G.L. writing – original draft preparation. O.G.L. writing – review and editing. F.M. visualization. O.G.L. supervision. O.G.L. project administration. O.G.L. funding acquisition. All authors have read and agreed to the published version of the manuscript.

## Funding

Open Access funding was enabled and organised by Projekt DEAL. The study was sponsored by MED‐EL Elektromedizinische Geräte GmbH, Innsbruck, Austria.

## Ethics Statement

All the procedures performed in studies involving human cadavers complied with the ethical standards of the institutional research committee and with the requirements of the current version of the Declaration of Helsinki.

## Consent

The authors have nothing to report.

## Conflicts of Interest

Francesca Maule, Pablo Galeazzi and Mohannad Al‐Qubaj are employees of MED‐EL Medical Electronics, Innsbruck, Austria. The other authors declare no conflicts of interest.

## Permission to Reproduce Material From Other Sources

The authors have nothing to report.

## Data Availability

The data that support the findings of this study are available from the corresponding author upon reasonable request.
